# Clinical and pathological analysis of solitary fibrous tumors with portal vein widening: A case report

**DOI:** 10.1097/MD.0000000000015757

**Published:** 2019-05-31

**Authors:** Xu-Qing Wang, Han-Qing Yang, Ji-Xiang Chen, Zhen-Fa Mao, He Han, Gong Chen, Xin Fan

**Affiliations:** Department of General Surgery, The Affiliated Hospital of Jiangsu University, Jiangsu Province, China.

**Keywords:** arteriovenous short circuit, portal hypertension, solitary fibrous tumors

## Abstract

**Rationale:**

Solitary fibrous tumors (SFTs) are rare soft-tissue tumors characterized with spindle-cell, which occur more common in the chest and rarely seen in the abdomen. So far as we knew, SFTs accompanied with venopathy of portal vein has rarely been reported.

**Patient concerns:**

A 36-year-old male presented with left-sided abdominal mass and portal vein expansion on ultrasound.

**Diagnoses:**

The post-operative histopathology confirmed the diagnosis of Solitary fibrous tumor.

**Interventions:**

Laparotomy was performed and the mass was completely removed.

**Outcomes:**

Patients had no symptoms, recovered well without recurrence; the portal vein and splenic vein dilatation were alleviated and the symptoms of portal hypertension were relieved.

**Lessons:**

SFTs presents with few symptoms in the early stage of the disease. A rich arteriovenous shunt is beneficial to the diagnosis of SFTs by B-ultrasound and computed tomography (CT) examinations. However, the diagnosis of SFTs must depend on histopathology.

## 1 Introduction

Solitary fibrous tumors (SFTs) were first proposed by Klemperer and Rabin in 1931.^[1]^ These tumors occur in various parts of the body. The onset of SFTs is insidious with no obvious symptoms at early stage.^[2]^ As a result, the diagnosis is somewhat difficult to ascertain at an early stage.^[3]^ For accurate results, the diagnosis of SFTs must always depend on histopathology.^[4]^ Early diagnosis of SFTs has proven to be helpful in both the treatment and prognosis of the condition. Currently, there are no effective methods to achieve an early diagnosis of SFTs. However, we found out from our study that a rich arteriovenous shunt is beneficial in diagnosing SFTs by b-ultrasound and computed tomography (CT) examinations. In our hospital, we recently admitted 1 case of abdominal SFTs with portal hypertension. Anatomical and pathological examination suggested that direct reflux of arterial blood draining into the vein through arteriovenous short circuit in the tumor contributed to portal vein disease. Subsequently, we retrospectively analyzed the clinical and pathological data of 12 patients with confirmed SFTs. Arteriovenous short circuit is a common pathological phenomenon in SFTs. We confirmed the manifestation on the basis of diversified literature and noticed that it presents with a series of symptoms related to hemodynamic changes.

## 2 Case presentation

A 36-year-old China male, who was previously healthy, was found to have a left-sided abdominal mass on physical examination. An abdominal ultrasound was preformed and revealed the presence of a large mass, The mass was located inferior to the kidney and measured, approximately 15.5 × 12.3 × 5.2 cm, The patient also had portal vein expansion (inner diameter of about 18 mm) a dilated the splenic vein (inner diameter about 16 mm). An abdominal CT examination confirmed the presence of a large and soft tissue mass in the left lower abdomen and spleen enlargement. CTA demonstrated that the tumor was located in the free abdomen below the left kidney with abundant blood supply and periphery in focus. The nourishing artery branched directly from the abdominal aorta under the celiac axis and the reflux vein was grossly distorted. The latter divided into 2 to 3 branches which drained into the portal vein posterior to the kidney. The portal vein was also widened. The branches of the splenic veins were distorted and expanded into the tumor.

Abnormalities were not seen in blood routine examination, biochemistry indexes, and the examination of coagulation routines. There were also no obvious abnormalities in the examination of electrocardiogram and chest radiograph.

Laparotomy was performed with general anesthesia and the mass was completely excised. In the operation, a solitary hemorrhagic tumor of 15.5 cm∗12.3 cm∗5.2 cm was found in the left lower abdomen. The surface of the mass was dark red and with a several large blood vessels. Most of the blood vessels were veins and the thickest one was about 1 cm in diameter. One of artery connecting to the abdominal aorta was considered as a nourishing artery according to CTA examination Several large distorted veins merged into the portal vein posterior of the kidney and spleen. The portal vein had a significantly wider lumen.

Its macroscopic inspection showed that the cut surface of the tumor was dark red with calcified areas. The nuclei of the tumors were markedly heteromorphic, with thick chromatin. The markers BCL-2 (+), CD34 (+), CD31 (+), and Vimentin (+), were positive, and the mitotic count was low. The proliferative index counted by Ki67 was weakly positive. Furthermore, elastic fiber staining demonstrated an arteriovenous short circuit inside the tumor. The final diagnosis for these manifestations was solitary fibrous tumor. Recovery was complete without a need for further treatment. The patient underwent abdominal ultrasound 10 months post-operation without recurrence, and the portal vein and splenic vein dilatation were alleviated (the inner diameter of the portal vein was about 17 mm). The symptoms of portal hypertension were also relieved.

## 3 Discussion

There are few special symptoms in the early stage of SFTs.^[5]^ The tumor usually becomes apparent when compression symptoms become apparent.^[6]^ There was no gender difference in SFTs. SFTs can occur almost anywhere in the body but is common in areas such as the pleura, pericardium, meninges, peritoneum, and so on.^[7]^ This patient had no clinical symptoms and was admitted for the mass and the wider portal vein (increased pressure) identified on physical examination. In this case, the feeder vessel of the tumor originated from the independent branch of the abdominal aorta, where pressure was higher; The vein directly returns to the portal vein. This anatomy contributed significantly to the increased portal vein pressure in this patient.^[8]^ Moreover, the capillary network connecting the artery and the vein could balance the difference in arteriovenous pressure. However, the blood supply of the case was not sufficient to explain the increased portal pressure (width widening). For this reason, we speculated that there may exist particular structures which could be responsible for the increased pressure of portal vein in the SFTs.

Ultrasound and X-ray can show the site of the lesion and morphological characteristics, but it is difficult to make a clear diagnosis. CT examination often reveals as isolated masses with clear boundaries and generally uniform density. If necrotic areas are present in the tumor, the masses may be uneven.^[9]^ On a general scale, enhanced scanning is significantly enhanced. However, certain differences can be noticed with different sites on the body. MRI can indicate the nature of lesions through signal characteristics and enhanced features.

A large number of studies have confirmed that SFTs can be classified as mesenchymal tumors. They are mainly composed of spindle cells and have a complex tissue structure, diverse cell morphology, and unclear boundaries.^[3]^ The cytoplasm is eosinophilic, the nucleus is irregular, and the chromatin distribution is uniform. The morphology of SFTs varies with the location. Tumor color is mostly grayish white the number of blood vessels. Texture of the tumor varies by the amount of collagen fibers present. The typical morphological feature of SFTs is the presence of interlaced dense and loose cells. Tumor cells in dense areas are arranged in bunches, vortexes, or irregular patterns, with obvious atypia, thick chromatin and uneven distribution of mitotic figures. The loose area is highly vascularized and is mainly composed of collagen.^[10]^

After a careful examination of routine sections, we found suspicious indication of arteriovenous shunts. To confirm the arteriovenous shunts, we used elastic fiber staining to distinguish arteries and veins, This helped us decipher whether or not an arteriovenous shunt exists in the solitary fibrous tumor. By considering the morphology of the tumor, radiographic findings, pathological results, and post-operative follow-up, it sufficed to conclude that:

1) the diagnosis of SFTs, in this case, is correct;
2) the pressure of feeder vessel is higher from the abdominal aorta.
3) the presence of arteriovenous shunts in the tumor leads to the increased pressure of the reflux vein, and the venous flow directly returns into the portal vein leading to an increased pressure in the portal vein.
4) the higher-pressure portal vein presents as wider inner diameter of the portal vein and slightly enlarged spleen on radiographic examination. Existing literature reports that solitary fibrous tumors have abundant blood supply.^[11]^

**Figure 1:**
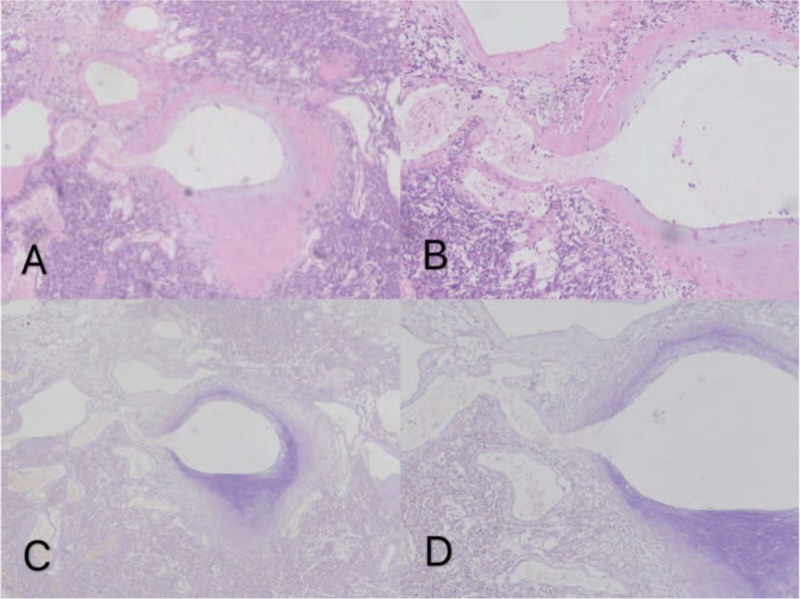
(A) The blood supply in the tumor is abundant, and multiple blood vessels are present (arrow). (HE staining ∗40) (B) The blood supply in the tumor is abundant, and multiple blood vessels are present (arrow). (HE staining ∗200) (C): Arterial wall significantly stained with elastic fibers, while negative staining (arrows) on the venous wall. (elastic fiber stained∗40) (D): The presence of traffic between the arteries and veins (arrows) can be clearly seen, and red blood cells remain in the traffic. (elastic fiber stained∗200).

**Figure 2:**
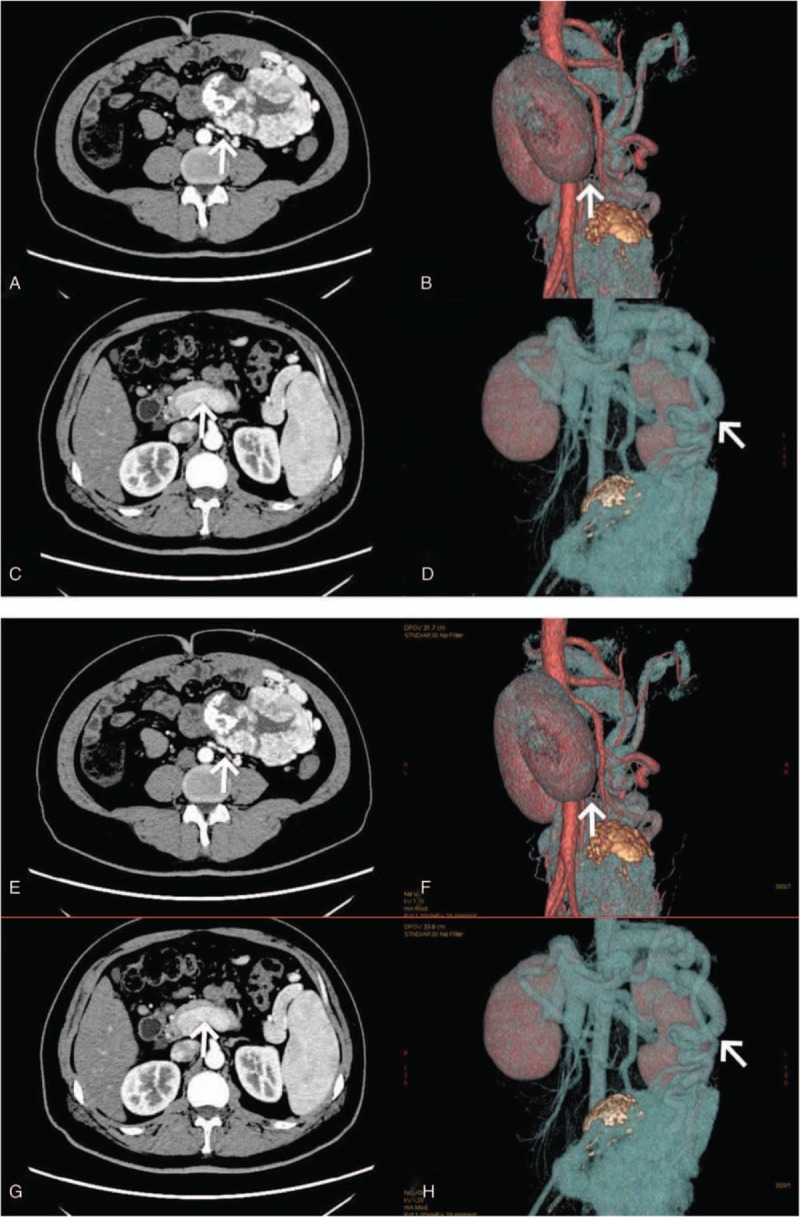
(A) The nourishing artery (arrow) of solitary fibrous tumors It is independently originated from the abdominal aorta, and nourishes the tumor. (abdominal CT scan) (B): The nourishing artery (arrow) of solitary fibrous tumors demonstrated with abdominal 3D revascularization. (C): The vein (arrow) of solitary fibrous tumors is located behind the kidneys and is divided into 2 branches which directly afflux to the portal vein. (abdominal CT scan)(D): The vein return (arrow) of solitary fibrous tumors showed by with abdominal 3D revascularization.

However, abnormal communication branches in SFTs have not been reported. The arteriovenous traffic of the tumor under microscope is shown in Figure 1. Vascular reconstruction of the tumor is shown in Figure 2.

Large, distorted blood vessels often present in SFTs, which is considered as a diagnostic criteria. Inspired by this case, we suspected that the wide and distorted vascular shadow in radiographic examination may cause increased intravascular pressure. Therefore, we retrospectively analyzed the other 12 patients (including 2 outpatients) who were diagnosed with solitary fibrous tumor by pathology in the past 3 years. We found arteriovenous shunts in 7/12 in the patients through the elastic fiber sections stained by paraffin. However, due to the incompleteness of clinical data, especially radiographic imaging in these cases, performing CTA reconstruction of tumor vessels was impossible. Therefore, relationship between large, distorted tumor blood vessels and arteriovenous shunts in tumor is still not clear. We proposed that in most cases arteriovenous shunts occur in solitary fibrous tumors, and can cause hemodynamic abnormalities such as portal hypertension. In addition, since not all the gross specimen is cut into sections for microscopic observation, arteriovenous shunts in the mass may be missed. Therefore, the actual incidence of arteriovenous shunts in SFTs may be higher than detected.

Currently, the markers commonly used to diagnose SFTs include CD34, CD31, cytokeratin, bcl-2, Calretinin, ki-67, p53, s-100, SMA, Vimentin, and wt-1 protein.^[12]^ As SFTs is derived from CD34 positive dendritic mesenchymal cells, CD34 is recognized as a relatively specific and accurate marker for SFTs. The CD34 positive detection rate of SFTs is close to 100%.^[13]^ However, positive CD34 is not unique to SFTs, and the diagnosis of SFTs should be combined with pathological morphological characteristics. The expression of p53, ki-67 was positively correlated with tumor malignancy.^[14]^ In this case, the pathological examination results showed typical morphological characteristics of SFTs, with obvious cellular atypia. Immunohistochemical results showed that CD34, CD31, and bcl-2 were strongly expressed and ki-67 was weakly positive. Therefore, it only requires combined analysis of with typical morphological and pathological examination as well as immunohistochemical detection to confirm SFTs.

The preferred treatment method for SFTs is surgery.^[15]^ Most benign tumors and some malignant tumors can be cured after surgical resection. Complete and/or incomplete resection of tumors closely determines the prognosis of SFTs.^[16]^ Adjuvant radiotherapy and chemotherapy can reduce the rate of postoperative recurrence and metastasis to a certain extent, which is often used for SFTs that is difficult to remove or has metastasized.^[17]^

## 4 Conclusions

SFTs are relatively rare diseases. When patients present with this disease, the arteriovenous shunts in the tumor is consider contributing significantly to the increased reflux venous pressure as well as portal and splenic venous pressure. We confirmed that arteriovenous shunts are a common phenomenon in the SFTs which could lead to a series of symptoms such as portal hypertension A rich arteriovenous shunt is a clue of SFTs diagnosis detected by B-ultrasound and CT examinations. But most importantly, the identification of SFTs must always depend on histopathological examination.^[18]^

## Author contributions

XF designed the work; XQW and HQY contributed to acquisition, analysis, or interpretation of data for the work; ZFM drafted the work; all authors contributed to the final approval of the version. We wish to thank our patient and his family for consenting to the publication of this case report.

**Conceptualization:** Xu-Qing Wang, Xin Fan.

**Data curation:** He Han, Gong Chen.

**Formal analysis:** Han-Qing Yang, Xin Fan.

**Funding acquisition:** Ji-Xiang Chen.

**Writing – original draft:** Han-Qing Yang, Zhen-Fa Mao.
